# Neural crest stem cells protect spinal cord neurons from excitotoxic damage and inhibit glial activation by secretion of brain-derived neurotrophic factor

**DOI:** 10.1007/s00441-018-2808-z

**Published:** 2018-03-07

**Authors:** Nikos Schizas, N. König, B. Andersson, S. Vasylovska, J. Hoeber, E. N. Kozlova, N. P. Hailer

**Affiliations:** 1 The OrthoLab, Department of Surgical Sciences, Section of Orthopaedics, Uppsala University, SE-751 85 Uppsala, Sweden; 2 Department of Neuroscience, Biomedicine Centre (BMC) Uppsala, BOX 593, SE-751 24 Uppsala, Sweden

**Keywords:** Neuroprotection, Suppressed glial activation, Excitotoxicity, Apoptosis, Secretion of soluble factors

## Abstract

The acute phase of spinal cord injury is characterized by excitotoxic and inflammatory events that mediate extensive neuronal loss in the gray matter. Neural crest stem cells (NCSCs) can exert neuroprotective and anti-inflammatory effects that may be mediated by soluble factors. We therefore hypothesize that transplantation of NCSCs to acutely injured spinal cord slice cultures (SCSCs) can prevent neuronal loss after excitotoxic injury. NCSCs were applied onto SCSCs previously subjected to *N*-methyl-d-aspartate (NMDA)-induced injury. Immunohistochemistry and TUNEL staining were used to quantitatively study cell populations and apoptosis. Concentrations of neurotrophic factors were measured by ELISA. Migration and differentiation properties of NCSCs on SCSCs, laminin, or hyaluronic acid hydrogel were separately studied. NCSCs counteracted the loss of NeuN-positive neurons that was otherwise observed after NMDA-induced excitotoxicity, partly by inhibiting neuronal apoptosis. They also reduced activation of both microglial cells and astrocytes. The concentration of brain-derived neurotrophic factor (BDNF) was increased in supernatants from SCSCs cultured with NCSCs compared to SCSCs alone and BDNF alone mimicked the effects of NCSC application on SCSCs. NCSCs migrated superficially across the surface of SCSCs and showed no signs of neuronal or glial differentiation but preserved their expression of SOX2 and Krox20. In conclusion, NCSCs exert neuroprotective, anti-apoptotic and glia-inhibitory effects on excitotoxically injured spinal cord tissue, some of these effects mediated by secretion of BDNF. However, the investigated NCSCs seem not to undergo neuronal or glial differentiation in the short term since markers indicative of an undifferentiated state were expressed during the entire observation period.

## Introduction

The acute phase of spinal cord injury (SCI) is characterized by a pathophysiological cascade of events that lead to neuronal damage through inflammatory and excitotoxic pathways (Gibson et al. 2004; Brown and Neher 2010; Freire 2012). Stem cell transplantation to the injured spinal cord has been extensively used in order to achieve exogenous reconnection (Dell’Anno and Strittmatter 2016) but it has also been recognized that transplanted stem cells can improve functional outcomes after SCI by other mechanisms than replacement of lost neurons (Mead et al. 2014; Gransee et al. 2015).

Neural crest stem cells (NCSCs) arise from the neural tube during early stages of embryonic development and develop into neurons and glia of the peripheral nervous system (Huang and Saint-Jeannet 2004). NCSCs are also present in bone marrow (Wislet-Gendebien et al. 2012) but the lack of specific markers that distinguish NCSCs from mesenchymal stem cells (Dominici et al. 2006) explains why these two types of stem cells are often commonly referred to as bone marrow stromal cells (BMSCs) (Neirinckx et al. 2014). NCSCs that are grafted to the lesioned spinal cord survive and express markers indicative of neuronal or oligodendrocytic differentiation (Sieber-Blum et al. 2006) and NCSCs transplanted to the site of a dorsal root avulsion injury can migrate to and undergo neuronal differentiation inside the spinal cord (Trolle et al. 2014; Konig et al. 2017).

The aim of the present study is to investigate whether acute application of boundary cap-derived NCSCs can modulate the extent of excitotoxic neuronal injury inside spinal cord tissue independent of later neuronal differentiation of the investigated stem cells. We hypothesize that application of NCSCs onto excitotoxically lesioned SCSCs would counteract the neuronal loss and perhaps mitigate the glial activation that is otherwise observed after excitotoxic injury to such cultures.

## Materials and methods

### Experimental design

All experiments were conducted under aseptic conditions using sterile instruments and after approval by the local ethics committee (C 346/11 Uppsala Ethical Committee on Animal Welfare). SCSCs were obtained from a total of 21 C57/Bl6 mice sacrificed on the ninth postnatal day (p9) and maintained on a hyaluronic acid hydrogel (Healon 5®; Abbott, Uppsala, Sweden).

#### Slice culture preparation

Mice were euthanatized by decapitation and the skin above the lower back was surgically removed to expose the lumbar and sacral regions. The spine was detached from the sacrum and a 23-gauge cannula was caudally inserted 2–3 mm inside the spinal canal. Subsequently, ice-cold preparation medium (MEM containing 1% glutamine, pH = 7.35) was injected through the cannula and the spinal cord was flushed out through the cervical spine. Using a tissue chopper (McIlwain Tissue Chopper; Mickle Laboratory Engineering, Surrey, UK), 500-μm slices were obtained from the cervical and lumbar regions and immediately transferred into Petri dishes containing preparation medium. The slices were placed in PET culture inserts coated with a layer of Healon 5® in the presence of SCSC medium consisting of 50% Minimal Essential Medium (MEM; Statens Veterinarmedicinska Anstalt (SVA), Uppsala, Sweden), 25% Hank’s balanced salt solution (HBSS; Gibco Life Technologies, Stockholm, Sweden), 25% normal horse serum (NHS; Gibco), 2% glutamine (Sigma-Aldrich, Stockholm, Sweden), 1 μg/mL insulin (Sigma-Aldrich), 2.4 mg/mL glucose (Sigma-Aldrich), 0.1 mg/mL streptomycin (SVA), 100 U/mL penicillin (SVA) and 0.8 μg/mL vitamin C (Sigma-Aldrich). The SCSCs were incubated at 35 °C in a 5% CO_2_-enriched atmosphere.

#### NCSC preparation

Primary cultures of NCSCs were prepared from transgenic heterozygous C57BL/6-A-actinY enhanced green fluorescent protein (eGFP) or DsRED mice according to previously published protocols (Hjerling-Leffler et al. 2005; Aldskogius et al. 2009). Dorsal root ganglia (DRGs) and dorsal and ventral roots were isolated from e11 embryos (Jackson Laboratories, Bar Harbor, Maine, USA) that showed expression of green or red fluorescent protein. The tissue was enzymatically dissociated using Collagenase/Dispase (1 mg/mL) and DNase (0.5 mg/mL) for 30 min at room temperature. Obtained cells were plated at 0.5–1 × 10^5^ cells/cm^2^ in N2 medium supplemented with B27 (Gibco, Grand Island, NY, http://www.invitrogen.com), epidermal growth factor (PeproTech, Rocky Hill, New Jersey, USA; 20 ng/mL) and basic fibroblast growth factor (bFGF; R&D Systems, Minneapolis, http://www.rndsystems.com; 20 ng/mL). On the subsequent day, non-adherent cells were removed. Half of the medium was changed every other day until neurospheres developed and the neurospheres were then kept free-floating in propagation medium (PROP: DMEM/F-12 medium (Invitrogen, 31330-038) supplemented with B27 (Invitrogen, 17504-044), N2 (Invitrogen, 17502-048) and containing 20 ng/mL bFGF (Invitrogen, 13,256–029) and 20 ng/mL EGF (R&D system, 236-EG)). Fifteen to 20 neurospheres containing a total number of approximately 10,000–20,000 cells each were transplanted onto the surface of SCSCs.

#### Exposure to NMDA and treatment groups

After 3 days in vitro (div), SCSCs were divided into four groups of which three were exposed to NMDA (50 mM; Sigma, Stockholm, Sweden) for 4 h. The rationale for performing a delayed excitotoxic injury was based on the previous observation that an immediate loss of neurons occurs early after culture preparation, a process that has stopped after 3 div (Schizas et al. 2013). The group that remained uninjured (no-NMDA group) served as a positive control, whereas one of the NMDA-injured groups remained otherwise untreated (NMDA group) and served as negative control. In the experimental group, NCSCs were applied on top of NMDA-injured SCSCs group 24 h after NMDA-induced injury (NMDA + NCSC group). An additional group was treated with IL1RA (IL1RA; Swedish Orphan Biovitrum (SOBi), Stockholm, Sweden; 1 μg/mL) directly after NMDA-induced injury since the neuroprotective effects of this treatment have been previously characterized (NMDA + IL1RA group) (Vogt et al. 2008; Schizas et al. 2014). A summary of the experimental groups together with the number of investigated cultures is given in Table 1.

**Table 1:** Experimental groups and number of SCSCs (*n*)

	Groups	Total *n*	Time of NCSC application	Endpoint	Analyses	*n* per group in each analysis
Excitotoxic injury of SCSCs and treatment with NCSC application	No-NMDA	30	4 div	10 div	NeuN	12
	NMDA	30			MacII	6
	NMDA + NCSC	30			GFAP	6
	NMDA + IL1RA	30			TUNEL	6
	No-NMDA	24	4 div	16 div		
	NMDA	24			NeuN	6
	NMDA + NCSC	24			MacII	6
	NMDA + IL1RA	24			GFAP	6
Migration of NCSCs on SCSCs	NCSCs on freshly prepared SCSCs	12	0 div	6 div	NeuN GFAP SOX2 Krox20	12
Release of neuroprotective factors from NCSCs on SCSCs	SCSCs + NCSCs SCSCs alone	9	0 div	6 h	ELISA	9
	SCSCs + NCSCs SCSCs alone	9	0 div	24 h	ELISA	9
BDNF as stand-alone treatment	No-NMDA	24	Not applied		NeuN	8
	NMDA			4 div		8
	NMDA + BDNF			10 div		8

**Fig. 1:**
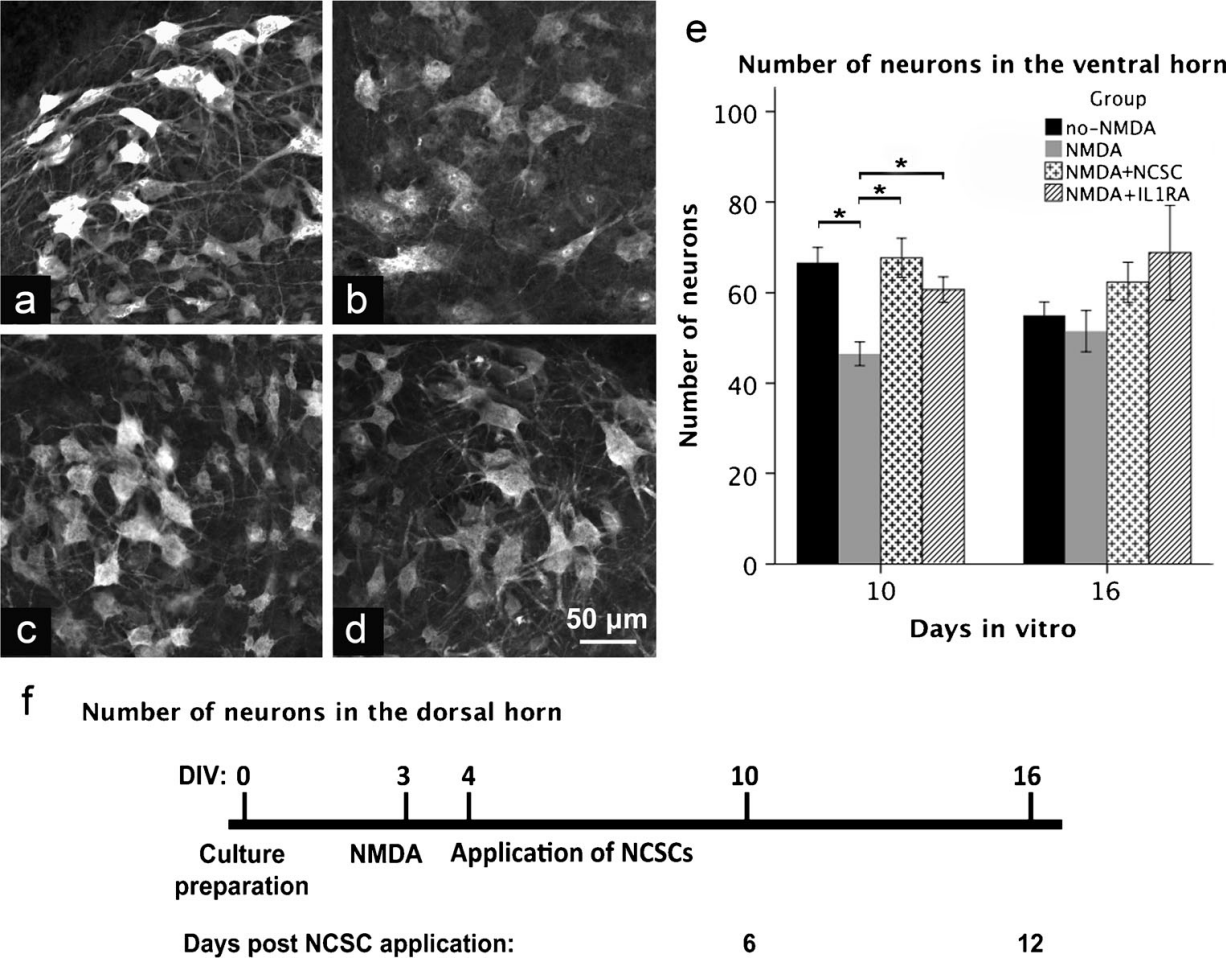
**a**–**f** Micrographs obtained through the ventral horn of SCSCs (**a** no-NMDA, **b** NMDA, **c** NMDA + NCSC, **d** NMDA + IL1RA) 10 days after the onset of the experiments using confocal microscopy after staining against NeuN. The diagram at the bottom (**f**) shows the timeline of the experiment. Graph (**e**) shows the number of counted neurons within the ventral horn in two different timepoints (bars denote means and spreads denote SEM)

SCSCs were maintained in vitro for a further 6 or 12 days after the application of NCSCs, i.e., for a total of 10 or 16 days after culture preparation (see Fig. 1 for a visualization of the time axis of all experiments). SCSCs were subsequently fixed using a mixture of paraformaldehyde and picric acid as described previously (Stavridis et al. 2005). Unsectioned SCSCs were stained for neuronal antigen (NeuN), Mac-II and glial fibrillary acidic protein (GFAP) in order to visualize neurons, activated microglial cells (Lalancette-Hebert et al. 2007) and astrocytes. Twelve to 18 SCSCs were obtained from each animal and each culture was considered an independent observation (*n*).

#### Identification of apoptotic cells

The TUNEL (TdT-mediated dUTP Nick End Labeling) assay was used in order to detect DNA strand breaks that are indicative of apoptosis. Apoptotic cells and apoptotic neurons were counted within the gray matter only since the white matter in SCSCs is susceptible to general degenerative changes as a consequence of in vitro culture. For the TUNEL-assays, six SCSCs from each group maintained for 6 days post-NCSC application were sectioned using a Leitz cryostat and stained for TUNEL, NeuN and DAPI as described below.

#### Migration of NCSCs on freshly prepared SCSC maintained in culture medium

In order to examine the migration and differentiation of NCSCs on SCSCs prior to scar tissue formation, a separate experiment based on NCSC application on freshly prepared SCSCs was performed. NCSCs were applied on top of freshly prepared SCSCs obtained from p9 mice, a drop of Healon 5® was applied onto the tissue complex containing SCSC and NCSC, thus creating a 3D matrix. These SCSCs were maintained for 6 div and subsequently fixed as described above. The SCSCs were sectioned longitudinally in order to study the migration pattern of NCSCs. In order to study the differentiation of NCSCs, longitudinal slides were stained for NeuN, GFAP, SRY [sex determining region Y]-box 2 (SOX2), a marker of multipotent neural stem cells (Ellis et al. 2004) and zinc-finger protein Krox20, a marker of early neural crest and neural crest boundary cap derived cells (Wilkinson et al. 1989). Krox20 has been reported to be a specific marker for boundary cap NCSC (Hjerling-Leffler et al. 2005; Oberlaender et al. 2009; Meyer et al. 2010). NeuN was chosen as a pan-neural marker since general neuronal survival was considered as the primary endpoint of the present study. Both cytoplasmic and nuclear presence of NeuN has been described previously (Oberlaender et al. 2009; Meyer et al. 2010; Dredge and Jensen 2011; Mead et al. 2014; Gusel’nikova and Korzhevskiy 2015; Kelly and Hawken 2017).

#### Migration of NCSCs into hyaluronic acid hydrogel (Healon 5®) in NCSC-differentiation medium

We performed the following set of experiments in order to study the migration of NCSCs in hyaluronic acid hydrogel (Healon 5®). Plain NCSC neurospheres were applied onto the biomaterial and maintained for 3, 6 and 10 days in the presence of NCSC-differentiation medium consisting of 1:1 DMEM/F12/Neurobasal supplemented with 1.2% non-essential amino acids (NEAA, 100×), 1% N2 (100×) and 1% B27 (50×; all from Invitrogen). Neurospheres maintained on standard laminin-coated coverslips in the presence of NCSC-differentiation medium were used as controls. Migration of NCSCs was studied using light and fluorescent microscopy.

### Immunohistochemistry and image analysis

Unsectioned SCSCs were washed in PBS for 30 min and incubated in 10% normal goat serum (Vector Laboratories, Järfälla, Sweden) for 60 min. All information about the primary antibodies including research resource identifiers (RRIDs) used in our study is presented in Table 2. Incubation with NeuN primary antisera (1:500, rabbit polyclonal, ABN78; Millipore, Temecula Billerica, CA, USA) for 24 h, or GFAP primary antisera (1:2000, AB7260, rabbit polyclonal; Abcam, Cambridge, UK) continued for 48 h. For microglia stains, cultures were incubated for 16 h with Mac-II–antibodies (1:400, CL8942F; BioSite, Stockholm, Sweden), either FITC-conjugated or biotin-conjugated and for biotin-conjugated Mac-II–antibodies were incubated with DyLight-549 for 2 h. SCSCs incubated with NeuN or GFAP primary antisera underwent a 2-h PBS washing and goat ant-rabbit secondary antibody (Vector Laboratories) followed for 16 h. After PBS washing, they were incubated with DyLight-488 (green) or DyLight-549 (red) Streptavidin (Vector Laboratory) for 2 h and washed for a further 2 h. When NCSCs were derived from eGFP-transgenic mice, DyLight-549 was used for counterstaining and when NCSCs were derived from DsRed-transgenic mice, DyLight-488 or FITC was used for this purpose. The SCSCs were then transferred to Super-Frost/Plus glass slides and mounted with Vectashield (Vector Laboratories).

**Table 2:** Primary antibodies

Primary antibody	Immunogen	Manufacturer, CN, RRID, host	Concentration
Anti-mouse NeuN	GST-tagged recombinant protein corresponding to mouse NeuN	Millipore Temecula CA USA, ABN78, AB_10807945, rabbit polyclonal	1:500
Anti-mouse MAC-II FITC or biotin conjugated	CLCR2A01, clone M3/38	Cederlane Burlington NC USA, CL8942F (or B), AB_10060162 (or AB_10059939), rat monoclonal	1:400
Anti-GFAP	Full-length human recombinant GFAP expressed in bacteria	Abcam Cambridge UK, ab7260, AB_305808, rabbit polyclonal	1:2000
Anti-SOX2	C terminus of human Sox-2	Santa Cruz Biotechnology, Inc. TX USA, sc-17,320, AB_2286684, goat polyclonal	1:100
Anti-Krox20/EGR2	Synthetic peptide located between aa397 and aa446 of human EGR2	LifeSpan Biosciences, Inc. WA USA, LS-B3577, AB_10627600, rabbit polyclonal	1:50

Images for subsequent analysis of NeuN-, Mac-II-, or GFAP-positive cells were captured using a Zeiss LSM 700 epifluorescence/confocal microscope. When DyLight-488, FITC, or eGFP fluorescence was used, images were scanned with a 488-nm laser and when DyLight-549 or DsRED fluorescence was used, images were scanned using a 543-nm laser. For NeuN-positive neurons, four images per culture were analyzed, two obtained from the ventral horns (one in each horn) and two from the dorsal horns (one in each horn). NeuN-positive neurons were counted automatically using the software CellProfiler (Carpenter et al. 2006) with an analysis pipeline recognizing neurons based on morphology, size and staining intensity. For analysis of MacII-positive microglial cells as well as GFAP-positive astrocytes, four images were obtained per culture, two from the median fissures representing parts of the white matter and two from parts within the gray matter. Activated microglial cells were counted separately within the gray and white matter using the same method and software as described above. GFAP-positive non-ramified astrocytes within the gray matter were manually counted using ImageJ software. The results presented express the number of cells per image.

### TUNEL assays

In order to count apoptotic cells within SCSCs, we used a TUNEL assay kit (TUNEL Enzyme CN: 1176705001 and TUNEL Label CN: 11767291910; Roche, Stockholm, Sweden) combined with immunohistochemistry for NeuN and DAPI. Six SCSCs from each group obtained 6 days after NCSC application (i.e., 10 days after culture preparation) were shock-frozen and sectioned at 20 μm using a Leitz cryostat. The sections were blocked with 10% normal goat serum and a TUNEL-fluorescein-labeled assay was performed according to the manufacturer’s protocol for 1 h at 37 °C. Incubation with NeuN primary antibody (1:500, rabbit polyclonal, ABN78; Millipore) was executed for 18 h at 4 °C. After washing, incubation with biotinylated goat anti-rabbit secondary antibody (Vector Laboratories) followed for 30 min and DyLight-549 streptavidin (Vector Laboratories) for another 30 min. DAPI solution (100 ng/mL; Life Technologies, Stockholm, Sweden) was then applied for 30 min and the sections were mounted with Vectashield.

Images were scanned using a Zeiss LSM 700 epifluorescence/confocal microscope with excitation wavelengths of 488 nm for fluorescein, 543 nm for DyLight-549 and 358 nm for DAPI. The obtained images were analyzed using CellProfiler software by splitting the images into three different color channels (red, NeuN; green, TUNEL; blue, DAPI). NeuN-immunoreactive neurons were counted using the image analysis algorithm described above under the “Immunohistochemistry and image analysis” section. TUNEL-positive and DAPI-positive nuclei were also counted using image analysis algorithms based on definitions of shape, color and staining intensity. In order to recognize apoptotic nuclei, a mask was created in order to examine overlap between structures. TUNEL-positive nuclei that overlapped with DAPI-positive nuclei were defined as apoptotic cells. TUNEL-positive nuclei that overlapped with DAPI-positive nuclei and furthermore overlapped with NeuN-positive neurons were defined as apoptotic neurons.

We used an index in order to examine the proportion of apoptotic cells in relation to NeuN-positive neurons. It was calculated as the fraction of the mean number of apoptotic cells over the mean number of all NeuN-positive neurons within the same area of the culture. The index was thus adjusted for general tissue degeneration since a degenerated culture would contain less neurons and more apoptotic cells.

### ELISA

The concentrations of brain-derived neurotrophic factor (BDNF), glial-derived neurotrophic factor (GDNF) and nerve growth factor (NGF) were examined in supernatants from SCSCs maintained in the presence or absence of NCSCs. SCSCs were obtained from p9 mice as described above, transferred into PET inserts (three cultures per insert) and hosted in culture wells containing culture medium. In contrast to the co-culture experiments described above, those designed for subsequent ELISA measurements were performed without the presence of Healon 5® since a considerable proportion of growth factors would have bound to this hyaluronic acid-based substrate, thereby inhibiting their release into the supernatant. NCSCs were applied onto the surfaces of SCSCs (SCSC + NCSC; *n* = 9) while an equal number of SCSCs without added NCSCs served as controls (SCSC alone; *n* = 9). Supernatants were collected from the culture wells after 6 and 24 h in sterile low-protein-binding tubes (VWR, Stockholm, Sweden) and immediately frozen at − 80 °C. ELISA was performed on the collected culture media using the Mouse-BDNF, Mouse-GDNF and Mouse-NGF ELISA Kits (Biomatic, CN: EKC36432, EKC36941 and EKC 37452). Standard concentrations in all samples were run in duplicate. Absorbance was measured on a Thermo Multiscan Ascent plate reader and the concentrations were calculated based on a standard curve that was generated using the Ascent Software for Multiscan.

We observed a statistically significant increase in the concentration of BDNF in NCSC-treated SCSC when compared to SCSCs not treated with NCSCs. Thus, an additional experiment was designed to investigate the effects of BDNF treatment on NMDA-lesioned SCSCs. Three groups were studied; untreated SCSCs (no-NMDA), excitotoxically lesioned SCSCs (NMDA) and excitotoxically lesioned SCSCs additionally treated with BDNF (NMDA + BDNF; *n* = 8 per group) in a concentration of 20 ng/mL (R&D Systems Bio-techne, UK). The cultures were studied 1 and 6 days after BDNF application (i.e., after 4 and 10 div, respectively). After fixation, the cultures were stained for NeuN as described above.

### Immunohistochemistry for NCSC markers

Longitudinal sections of SCSCs with transplanted NCSCs were pre-incubated with blocking solution (1% bovine serum albumin, 0.3% Triton X-100 and 0.1% sodium azide (NaN3) in PBS) for 45 min at room temperature and then incubated overnight at 4 °C with primary antibodies for SOX2 (1:100, sc-17320; Santa Cruz Biotechnology, Santa Cruz, USA) or Krox20 (1:50, LS-B3577; BioSite, Stockholm, Sweden). Sections were washed with PBS and incubated with secondary antibodies (Alexa 555) in blocking solution for 1 h at room temperature. After removing the secondary antibodies, sections were washed with PBS and embedded in mounting medium (50% glycerol in PBS and 100 mM propyl-gallate; Sigma).

### Randomization and blinding

The SCSCs were randomly assigned to each treatment group by a laboratory assistant who was blinded to the treatment planned for each culture. SCSCs from different animals were assigned to each experimental group, thus ensuring that cultures from different animals were evenly represented in the different experimental groups. SCSCs treated by NCSC transplantation were randomly chosen and application of NCSCs was performed by an independent laboratory assistant. The application was performed using a microscope in order to ensure that a standard number of 15–20 neurospheres would be applied to the surface of each SCSC.

During microscopic analysis, the observers could distinguish the group treated with NCSC application through the autofluorescence of NCSCs; therefore, blinding was impossible with respect to this specific aspect. In order to reduce potential bias, a double-observer analysis with a second independent observer was performed. The inter-observer coefficient of variation was defined as the standard deviation of the inter-observer difference divided by the mean of all samples and was found to be 12%.

Image analysis was performed automatically using the CellProfiler software with an analytical pipeline that remained unaltered during all analyses.

### Statistical analysis

Normality of data distributions was investigated using the Kolmogorov–Smirnov test and the assumption of equal variances between groups was investigated using Levene’s test. For statistical analysis of NeuN- and Mac-II-positive cells, a one-way ANOVA with planned contrasts was applied for comparisons of the various experimental groups (no-NMDA, NMDA, NMDA + NCSC, NMDA + IL1RA). For the analysis of GFAP-positive cells, a one-way ANOVA followed by a Bonferroni correction was applied. In the analysis of TUNEL assays, data distributions within groups deviated significantly from normality and thus non-parametric Kruskal–Wallis tests followed by Mann–Whitney *U* tests were applied. The level of statistical significance was set at 0.05 and all data are presented as means ± standard error of the mean (SEM).

## Results

### NCSCs protect ventral horn neurons from excitotoxic damage

Ten days after culture preparation, excitotoxically injured SCSCs treated with NCSC transplantation contained more NeuN-positive neurons in the ventral horns (67.6 ± 4.3) than excitotoxically injured SCSCs not treated with stem cell transplantation (46.5 ± 2.5, *p* = 0.001, Fig. 1). The number of preserved neurons in transplanted SCSCs was thus comparable to the number of NeuN-positive neurons counted in uninjured SCSCs (66.7 ± 3.3). IL1RA also counteracted the neuronal loss observed after excitotoxic injury with NMDA (60.7 ± 2.8, *p* = 0.002 compared to the NMDA group). However, 16 days after culture preparation, no significant differences were noted between the experimental groups. Within the dorsal horn, no significant differences were noted in the number of NeuN-positive neurons between groups, neither 10 nor 16 days after culture preparation.

### The proportion of apoptotic cells decreases after stem cell transplantation

**Fig. 2:**
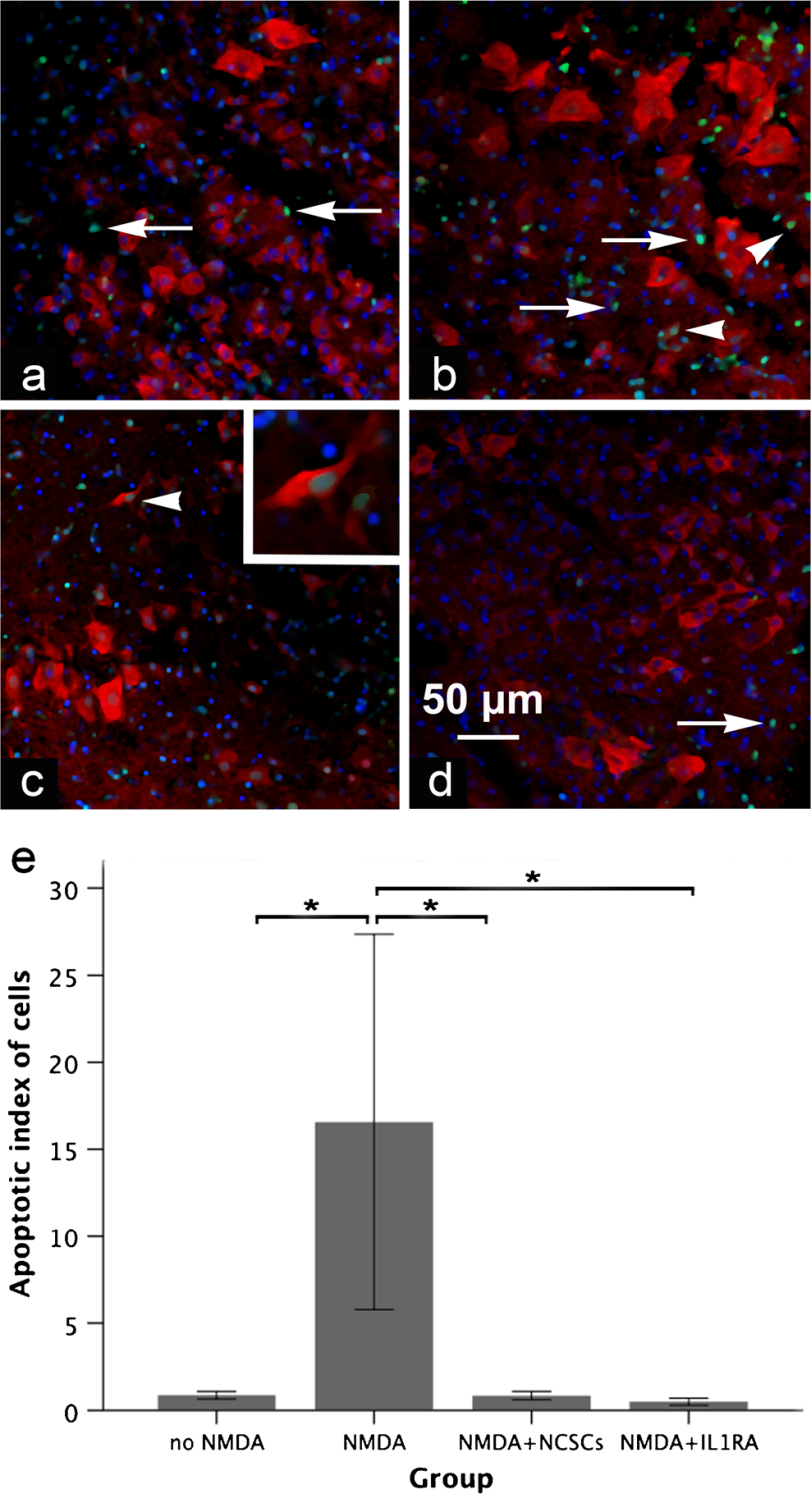
**a**–**e** Micrographs of sections through the gray matter of SCSCs (**a** no-NMDA, **b** NMDA, **c** NMDA + NCSC, **d** NMDA + IL1RA) 6 days post-NCSC application stained for NeuN (red), TUNEL (green) and DAPI (blue). The inner parts of the sections were devoid of e-GFP-positive NCSCs since the latter remained on the surface of the slice cultures. The above sections were obtained from the inner parts of the cultures, an area that NCSCs did not reach. Apoptotic cells were identified by co-localization of TUNEL and DAPI (arrows) and apoptotic neurons were identified as co-localization of TUNEL, DAPI and NeuN (arrowheads, shown also in higher magnification in the right top corner in **c**). The proportion of apoptotic cells in relation to the number of neurons was significantly higher in the NMDA group (**b**) compared to the other groups. Graph (**e**) shows the apoptotic index of cells in different groups (statistical analysis was performed by non-parametric Kruskal–Wallis followed by Mann–Whitney *U* tests; solid bars indicate means and error bars denote SEM, *p* ≤ 0.05)

Transplantation of NCSCs onto excitotoxically injured SCSCs prevented the increase in apoptotic neurons that was observed in injured SCSCs not treated with stem cells (apoptotic index for cells—0.5 ± 0.2 in the NMDA + NCSC group compared to 16.6 ± 10.8 in the NMDA group, *p* = 0.008, Fig. 2). In the uninjured control group, the apoptotic index was 0.9 ± 0.2 and treatment of NMDA-injured SCSCs with IL1RA also reduced the proportion of apoptotic cells among neurons compared to the NMDA group (apoptotic index for cells, 0.4 ± 0.1; *p* = 0.016, Fig. 2).

### Activated microglial cells are less numerous in white matter after stem cell transplantation

**Fig. 3:**
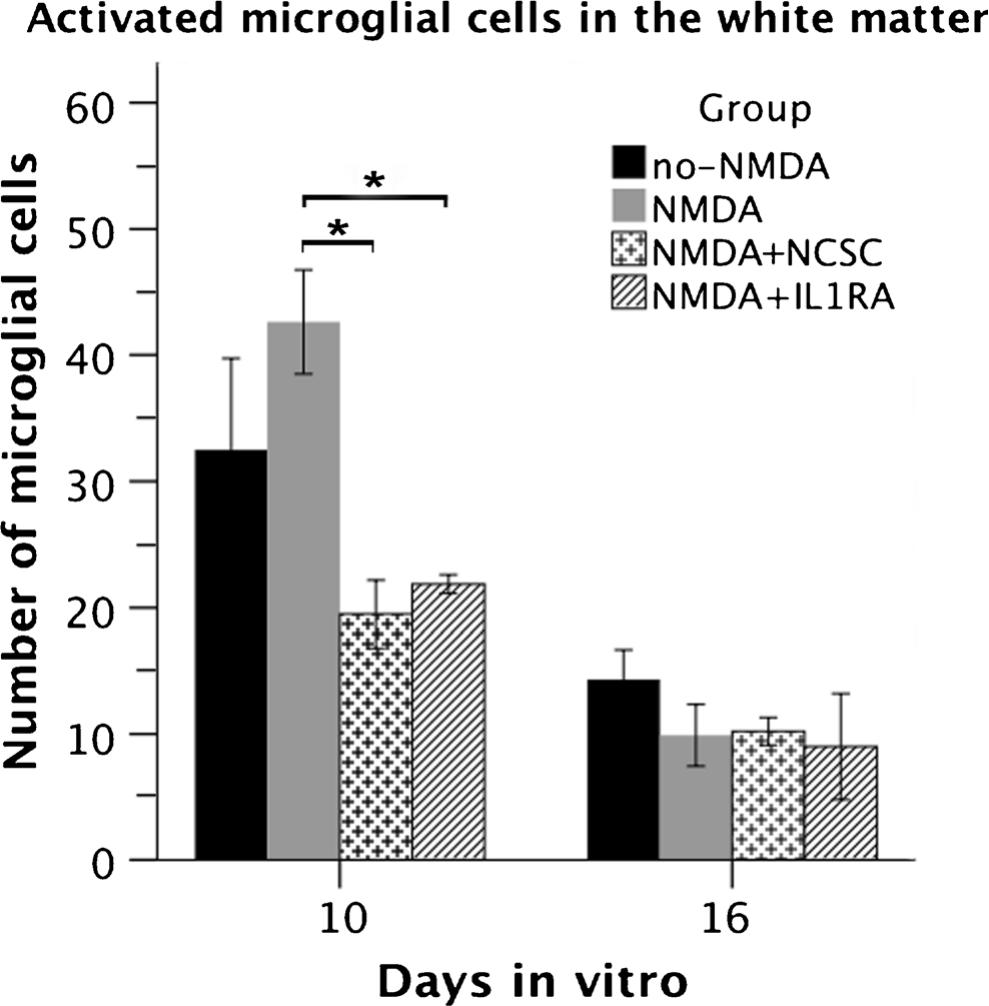
The graph shows the number of activated microglial cells within the white matter of SCSCs in two different timepoints (statistical analysis performed by ANOVA with planned contrasts; solid bars indicate means and error bars denote SEM, *p* ≤ 0.05)

Transplantation of NCSCs onto excitotoxically lesioned SCSCs reduced the number of activated microglial cells in the white matter (19.5 ± 2.7 in the NMDA + NCSC group compared to 42.6 ± 4.1 in the NMDA group; *p* = 0.004, Fig. 3). IL1RA also reduced the number of activated microglial cells in the white matter to 21.9 ± 0.7 (*p* = 0.01 compared to NMDA group, Fig. 3). Sixteen days after culture preparation, no relevant differences in the number of activated microglial cells in the white matter were noted between the groups.

In the gray matter, a very small number of activated microglial cells was observed both 10 and 16 days after the onset of the experiments but that number did not differ statistically significantly between the groups.

### Non-ramified astrocytes in the gray matter are less numerous in the group treated with NCSCs

**Fig. 4:**
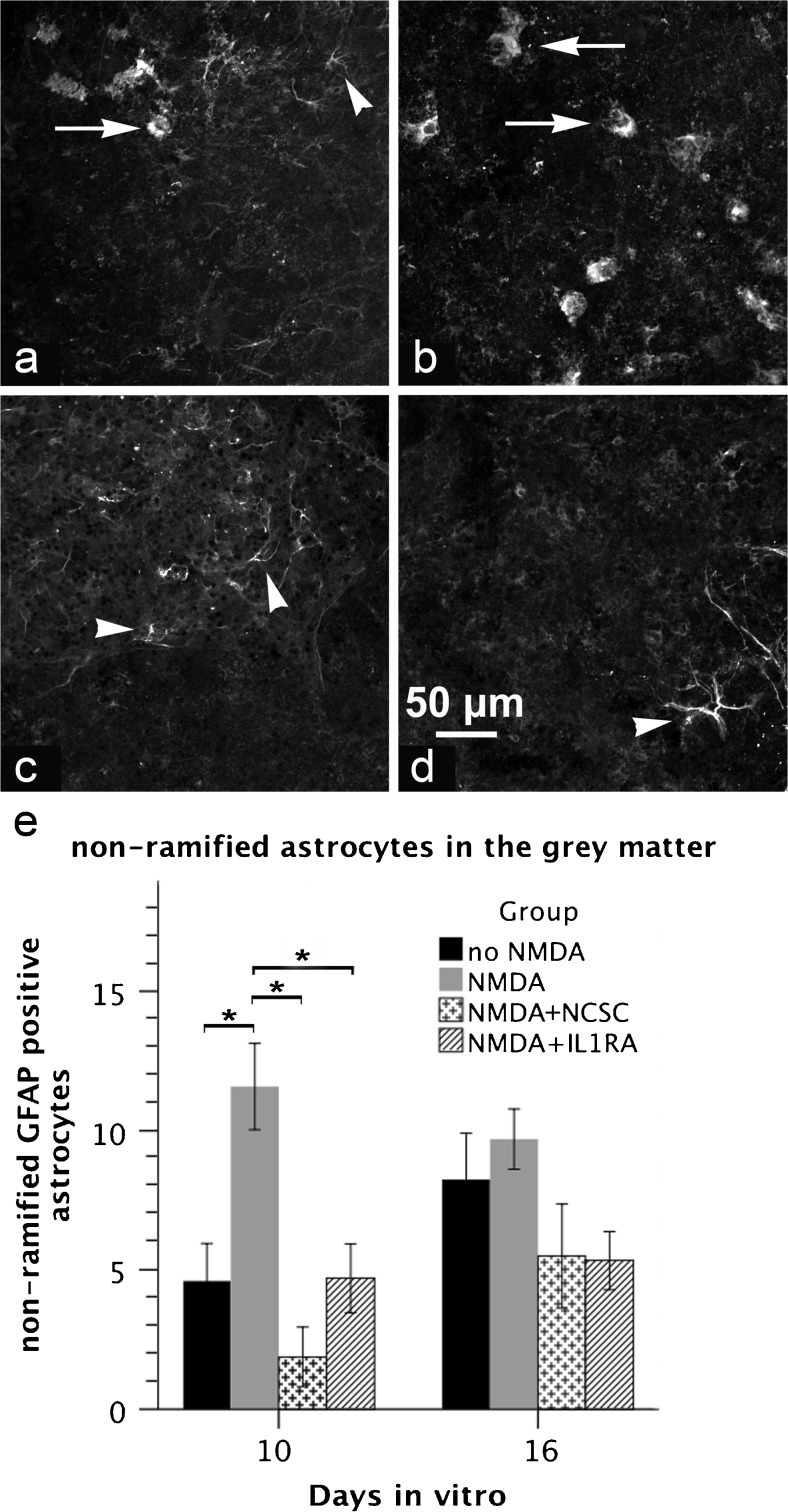
**a**–**e** Micrographs obtained through the gray matter of SCSCs (**a** no-NMDA, **b** NMDA, **c** NMDA + NCSC, **d** NMDA + IL1RA) 10 days after the onset of the experiments using confocal microscopy after staining against GFAP. In order to examine the effect of NCSCs on astroglial activation, non-ramified GFAP-positive astrocytes were counted within the gray matter. Arrows denote non-ramified activated astrocytes, while arrowheads denote astrocytes in a resting state. Graph (**e**) shows the number of non-ramified GFAP positive astrocytes within the gray matter of SCSCs in two different timepoints (statistical analysis was performed by ANOVA followed by Bonferroni correction; solid bars indicate means and error bars denote SEM, *p* ≤ 0.05)

Application of NCSCs significantly reduced the number of non-ramified GFAP-positive astrocytes in the gray matter of excitotoxically lesioned SCSCs (1.9 ± 1.1 in the NMDA + NCSC group compared to 11.6 ± 1.5 in the NMDA group, *p* = 0.001, Fig. 4). Treatment with IL1RA also reduced the number of non-ramified astrocytes after NMDA induced injury to 4.7 ± 1.2 (*p* = 0.01 vs. the NMDA group).

Within the white matter, astrocytes had phenotypes with slender bodies and fine ramifications and there were no obvious differences in the morphology of white-matter astrocytes when comparing the different experimental groups.

### The concentration of BDNF is higher in supernatants from SCSCs treated with stem cell transplantation and BDNF alone also protects ventral horn neurons from excitotoxic injury

**Fig. 5:**
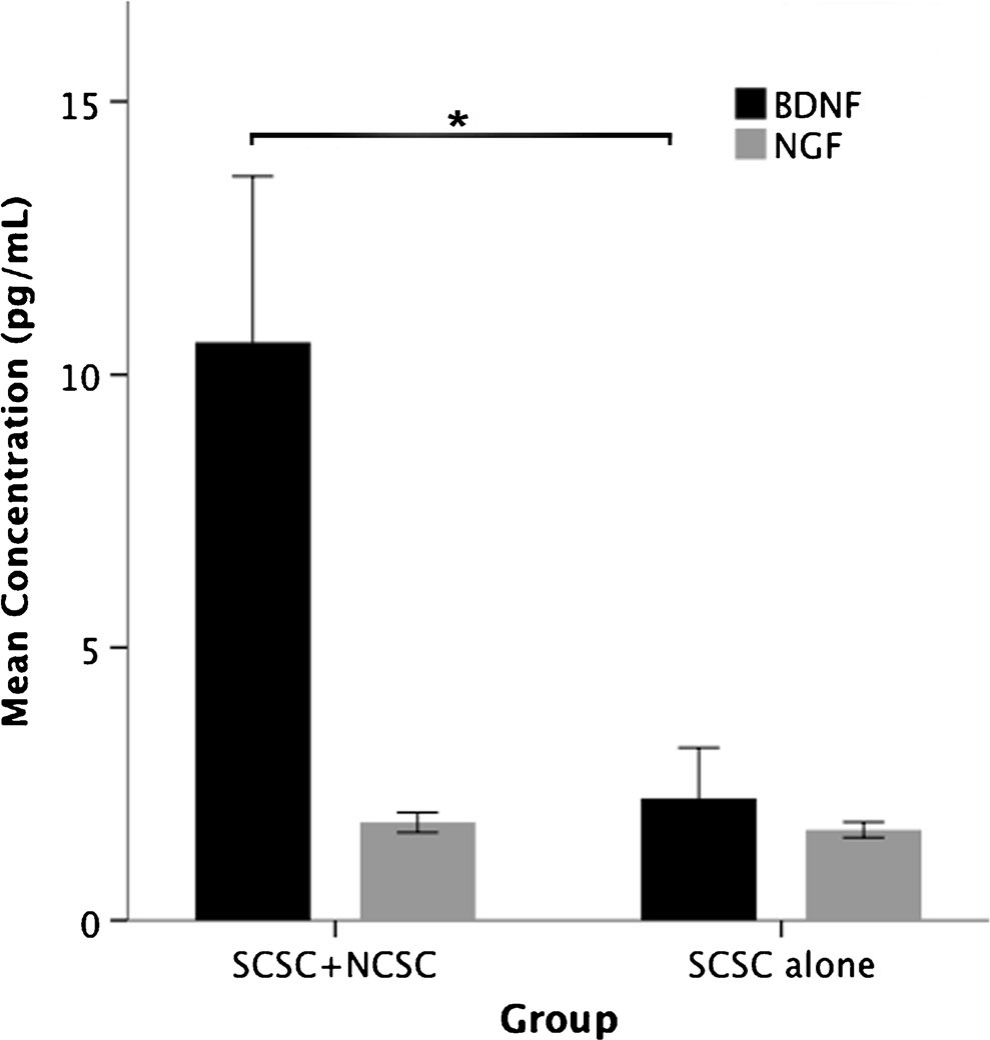
The graph shows the concentrations of BDNF and NGF in supernatants from co-cultures (SCSCs and NCSCs) and SCSCs alone after a 24-h incubation. The concentration of BDNF was significantly higher in the SCSC–NCSC group compared to SCSC alone (statistical analysis was performed by ANOVA; solid bars indicate means and error bars denote SEM, *p* ≤ 0.05)

Twenty-four hours after transplantation of NCSCs to SCSCs, the concentration of BDNF in culture supernatants was higher when compared to the supernatant from SCSCs that were not treated by stem cell transplantation (10.6 vs. 2.2 pg/mL, *p* = 0.025; Fig. 5). In contrast, the concentration of NGF in supernatants did not differ between these two groups. There were also no statistically significant differences between the two investigated groups when measuring the concentrations of BDNF or NGF after 6 h and GDNF was not found at measurable concentrations in supernatants from any culture at any investigated time point.

BDNF was additionally used as stand-alone treatment of SCSCs subjected to excitotoxic injury, without any stem cell transplantation. After 10 div (i.e., 6 days post-BDNF application), application of BDNF without additional stem cell treatment counteracted the neuronal loss seen in NMDA-lesioned SCSCs (NMDA + BDNF, 59.3 ± 2.5 vs. 33.4 ± 4.4 in NMDA-lesioned cultures; *p* < 0.001). For comparison, the number of neurons in the ventral horn of uninjured cultures was 55.6 ± 2.9 (*p* < 0.001 when compared with NMDA-lesioned cultures).

### Migration of NCSC across the surface of SCSCs and within the hydrogel in the presence of culture medium

**Fig. 6:**
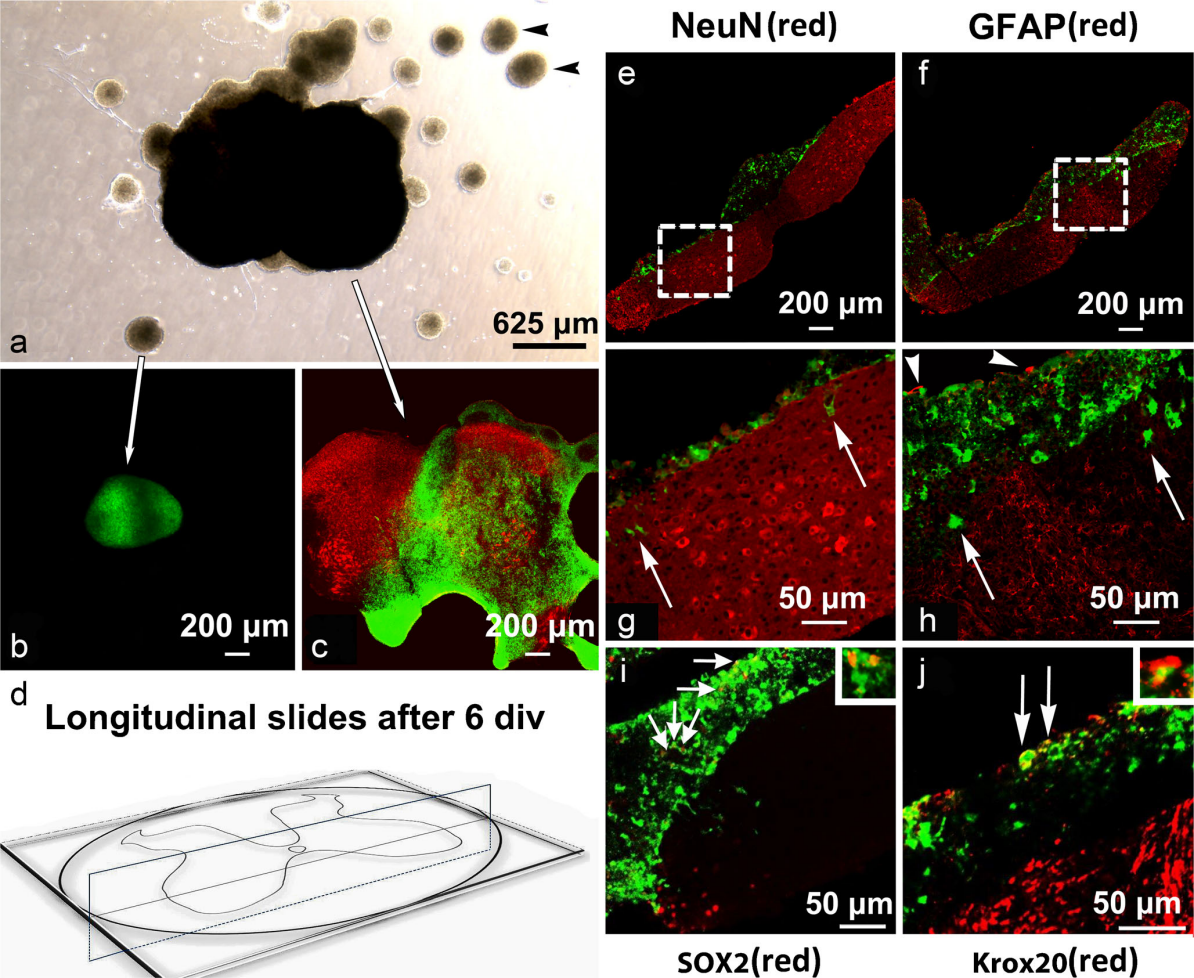
**a**–**j** Images of SCSCs maintained for 6 days in vitro after culture preparation and immediate application of NCSCs. Images of unsectioned cultures and neurospheres after light and confocal microscopy (**a**–**c**): black arrowheads in image of light microscopy (**a**) show neurospheres of NCSCs with no signs of migration. Confocal laser scanning images (**b**) and (**c**) show NCSCs marked with eGFP (green) and NeuN (DyLight-549, red) after immunohistochemistry. NCSCs migrated on top of SCSCs (**c**) but remained in spheres when there was no contact with the cultures (**b**). Images of longitudinal sections through SCSCs and NCSCs (**e**, **f**, **g**, **h**). Diagram (**d**) shows how the cultures were sectioned longitudinally. NCSCs were applied on SCSCs directly after culture preparation in order to avoid scar formation that occurs on top of the cultures and maintained for 6 days in vitro. Images (**g**) and (**h**) represent parts of (**e**) and (**f**) in a higher magnification indicated by dashed lines. The majority of NCSCs migrated on the surface of the culture and some NCSC appeared to migrate through the culture (white arrows). The spheres stained against GFAP showed signs of GFAP immunoreactivity in the edges (white arrowheads in **h**) but no signs of co-localization between GFAP and eGFP autofluorescence were observed, suggesting astrocytic migration from SCSCs through the neurospheres. Staining of longitudinal sections against SOX2 and Krox20 (**i**, **j**). Few cells showed colocalization with SOX2 (small arrows in **i**) and Krox20 (large arrows in **j**), markers of undifferentiated neural crest stem cells. The small boxes in the upper right-hand corner of (**i**) and (**j**) represent areas of co-localization between eGFP and SOX2 or Krox20, respectively, in higher magnification

NCSCs originating from neurospheres that were in contact with SCSCs migrated across the surface of SCSCs (Fig. 6a–c) but only a few NCSCs migrated deeper into the SCSC tissue. No migration of NCSCs was noted from neurospheres that were not in contact with SCSCs (Fig. 6a–c). In order to further investigate the ability of NCSCs to migrate into SCSCs, we performed experiments using freshly prepared SCSCs that had not yet undergone scar formation. Analyses of longitudinal sections obtained after 6 div confirmed that the majority of NCSCs did not migrate into the cultures but remained on the surface. Very few NCSCs were found within the SCSCs and in those few cases they remained close to the surface of the cultures (Fig. 6g, h). Staining of longitudinal sections through tissue cultures did not reveal NeuN or GFAP immunoreactivity on eGFP-expressing NCSCs (Fig. 6e, f), whereas some eGFP-expressing NCSC showed immunoreactivity for SOX2 and Krox20 (Fig. 6i, j).

**Fig. 7:**
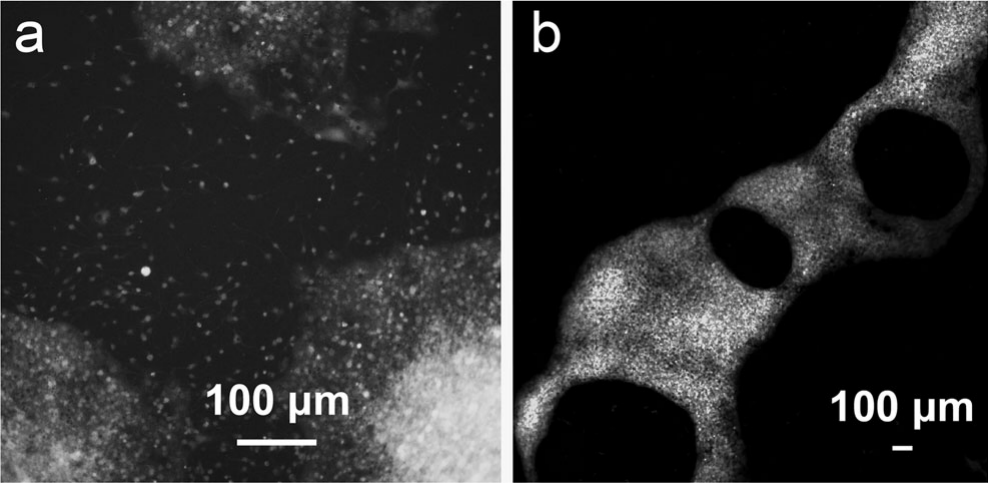
**a**, **b** Overview images of neurospheres maintained on laminin-coated coverslips (**a**) and Healon 5® (**b**) after 3 div in the presence of NCSC-differentiation medium. NCSCs from all the neurospheres maintained on standard laminin-coated coverslips migrated over the area of the coverslip after 3 div in contrast to neurospheres maintained on Healon 5®. Migration of NCSCs from neurospheres maintained on Healon 5® was observed in only 12% of neurospheres after 3 div and in 25% of neurospheres after 6 div

The migratory behavior of NCSCs was further investigated on different substrates. Neurospheres that were maintained on laminin-coated coverslips in the presence of differentiation medium flattened out after 3 div and migration of NCSCs across the laminin surface regularly occurred (Fig. 7). In contrast, migration of NCSCs outside neurospheres was only infrequently observed when maintained on hyaluronic acid hydrogel.

## Discussion

### NCSCs protect spinal cord neurons from excitotoxic damage

The present study demonstrates that transplantation of boundary-cap derived NCSCs onto cultured spinal cord tissue that has previously been subjected to NMDA-induced excitotoxicity exerts neuroprotective effects on NeuN-positive neurons in the ventral horns. NCSC application reduces the number of activated microglial cells and counteracts astrocytic activation. Our findings indicate that such stem cells could potentially be useful in the acute phase after SCI, not by neuronal differentiation and substitution of apoptotic neurons but by supporting neuronal survival and by inhibiting apoptosis and gliosis.

A range of neurotrophic and angiogenic factors are secreted by NCSCs in isolated cultures, including BDNF, GDNF, NGF and neurotrophins (NT) 3 and 5 (Lau et al. 2015b). In the present study, the concentration of BDNF was higher in culture supernatants from SCSCs transplanted with NCSCs compared to SCSCs without additional stem cell treatment. Moreover, treatment of NMDA-lesioned SCSCs with BDNF as a stand-alone treatment supported neuronal survival within the ventral horn to a similar extent as stem cell treatment. These findings suggest that BDNF may be an important mediator of the neuroprotective effects induced by the NCSCs. This interpretation is supported by the fact that BDNF is known to regulate and reduce toxic NMDA-receptor signaling, thus protecting from excitotoxic damage (Lau et al. 2015a).

Having said that, although NCSCs are a probable origin of BDNF in our transplantation experiments, our findings leave room for the interpretation that the presence of stem cells induces other cells residing in the SCSCs to produce this growth factor. We found no measurable concentrations of NGF in our supernatants, although release of this factor from mesenchymal stem cells is described (Crigler et al. 2006).

### NCSC transplantation reduces the proportion of apoptotic cells

We found a reduction in the proportion of apoptotic cells in stem cell-transplanted, excitotoxically lesioned cultures that coincided with increased survival of viable neurons. Other types of stem cells, such as BMSCs, have also been associated with anti-apoptotic effects following SCI, probably by down-regulation of caspase-3 (Dasari et al. 2007; Lin et al. 2013). Anti-apoptotic strategies include antioxidants and inhibition of 12-lipoxygenase, inactivation of caspases and application of neurotrophic factors (Okouchi et al. 2007). NCSCs might possess anti-apoptotic properties through the release of such anti-apoptotic agents and subsequent interruption of apoptotic pathways.

### NCSCs suppress micro- and astroglial activation

The assumption of anti-inflammatory actions of NCSCs on activated microglial cells is strengthened by the fact that NCSCs are protective following cytokine-induced cell death (Ngamjariyawat et al. 2012; Ngamjariyawat et al. 2013). SCSCs exposed to NMDA showed an increased number of non-ramified GFAP-positive astrocytes within the gray matter compared to uninjured SCSCs but transplantation of NCSC onto injured SCSCs reduced the number of these presumably activated glial cells.

The fact that NCSC application was able to reduce the number of activated microglial cells in the white matter, i.e., a topographical structure that does not contain neurons, indicates direct effects of NCSCs on microglia. Astrocytes, on the other hand, had non-activated phenotypes in the white matter in SCSCs, an observation that is in contrast to the microglial activation found within the white matter of such cultures. A possible explanation is that astrocytes do not respond to signaling coming from the white matter to the same extent as microglial cells, at least within the relatively short-time frames investigated within our study. However, astrocytes seem to respond to signaling coming from the gray matter (i.e., from neurons subjected to excitotoxic damage) by altering their state of activation (Hailer et al. 1999). Thus, it seems reasonable to assume that NCSCs indirectly suppress astrocytic activation by counteracting excitotoxicity.

The spatiotemporal pattern of astrocyte activation at earlier timepoints was also studied (data not shown). We observed a delayed astrocytic activation in terms of retraction of branches that was present after 7–8 div. We therefore suggest that neuronal degeneration and microglial activation early during in vitro culture can occur regardless of astrocytic activation.

It must be considered that glial activation may be beneficial at later stages after SCI (Rolls et al. 2008; White et al. 2011). Therefore, modulatory effects of stem cell treatment on glial activation cannot by default be considered desirable and long-term experiments on the interaction of transplanted stem cells with the glial population are required. It is also obvious from previous studies that transplanted stem cells can differentiate to glial cells to a relatively large extent (Trolle et al. 2014; Konig et al. 2017), indicating that stem-cell derived glial cells can exert a multitude of supportive effects on both resident and stem-cell derived neurons.

### Migration of NCSCs

When transplanted neurospheres were in contact with SCSCs, migration of NCSCs across the culture surface occurred. However, NCSCs did not migrate into the underlying tissue, even when NCSCs were applied onto freshly prepared SCSCs that had not yet undergone scar formation. Thus, scar tissue formation along the surface of SCSCs that inevitably occurs during in vitro culture seems not to be the reason for the observed inability of NCSCs to migrate into SCSCs. This observation is partly consistent with previous studies concerning application of NCSCs on pancreatic islets (Grouwels et al. 2012; Lau et al. 2015b). NCSCs migrated across the surface of pancreatic islets, although some cells migrated within the islets. Dendritic and axonal extensions originating from NCSCs were observed within pancreatic islets transplanted with NCSCs, suggesting that these extensions could represent the portals for delivery of protective factors.

It thus seems that SCSCs provide a suitable substrate for NCSC migration; however, signaling to initiate NCSC differentiation seems to be absent or suppressed. In contrast, NCSCs maintained on laminin-coated coverslips in the presence of NCSC differentiation media showed signs of glial and neural differentiation already after 3 days in vitro.

Epidermal NCSCs that were transplanted into the lesioned spinal cord did not migrate from the transplantation site after 2 to 4 months (Sieber-Blum et al. 2006). Although the above observation is in accordance with ours, NCSCs derived from the boundary cap and transplanted into lesioned spinal cord did migrate and these stem cells also showed signs of both neural and glial differentiation (Zujovic et al. 2010; Trolle et al. 2014). Differences between boundary cap-derived NCSCs and epidermally derived NCSCs were recently demonstrated in a comparative study (Kosykh et al. 2014) and these could explain the differences in migratory behavior. NCSC differentiation media had detrimental effects on the survival of SCSCs (data not shown), thus, experiments on the ability of NCSC to differentiate when in contact with both spinal cord tissue and suitable differentiation media could unfortunately not be performed.

## Conclusions

NCSCs derived from the boundary cap have neuroprotective and anti-apoptotic effects on spinal cord neurons exposed to excitotoxic damage. These stem cells did not enter the spinal cord tissue but, since application of BDNF alone was able to mimic some of the effects of NCSCs, it seems probable that NCSCs exert some of their effects by secretion of BDNF. Suppressed activation of both microglial cells and astrocytes may be the result of direct anti-inflammatory effects of NCSC on the glial population but these findings have to be investigated further and inhibition of glial cells may not be a desirable effect at later stages after SCI. Since NCSCs were unable to migrate or differentiate within a hyaluronic acid hydrogel, the choice of other suitable biomaterials is critical if incorporation of NCSCs into carriers is considered.

### Support and grant information

The study was funded by the regional agreement on medical training and clinical research (ALF) between Uppsala County Council and Uppsala University, by the Swedish Research Council (20716 to E.N.K.) and by Stiftelsen Olle Engkvist Byggmästare.
